# The Human-Baited Double Net Trap: An Alternative to Human Landing Catches for Collecting Outdoor Biting Mosquitoes in Lao PDR

**DOI:** 10.1371/journal.pone.0138735

**Published:** 2015-09-18

**Authors:** Julie-Anne A. Tangena, Phoutmany Thammavong, Alexandra Hiscox, Steve W. Lindsay, Paul T. Brey

**Affiliations:** 1 Department of Entomology, Institut Pasteur du Laos, Vientiane, Lao PDR; 2 School of Biological and Biomedical Sciences, Durham University, Durham, United Kingdom; 3 Laboratory of Entomology, Wageningen University and Research Centre, Wageningen, the Netherlands; New Mexico State University, UNITED STATES

## Abstract

Estimating the exposure of individuals to mosquito-borne diseases is a key measure used to evaluate the success of vector control operations. The gold standard is to use human landing catches where mosquitoes are collected off the exposed limbs of human collectors. This is however an unsatisfactory method since it potentially exposes individuals to a range of mosquito-borne diseases. In this study several sampling methods were compared to find a method that is representative of the human-biting rate outdoors, but which does not expose collectors to mosquito-borne infections. The sampling efficiency of four odour-baited traps were compared outdoors in rural Lao PDR; the human-baited double net (HDN) trap, CDC light trap, BG sentinel trap and Suna trap. Subsequently the HDN, the best performing trap, was compared directly with human landing catches (HLC), the ‘gold standard’, for estimating human-biting rates. HDNs collected 11–44 times more mosquitoes than the other traps, with the exception of the HLC. The HDN collected similar numbers of *Anopheles* (Rate Ratio, RR = 1.16, 95% Confidence Intervals, 95% CI = 0.61–2.20) and *Culex* mosquitoes (RR = 1.26, 95% CI = 0.74–2.17) as HLC, but under-estimated the numbers of *Aedes albopictus* (RR = 0.45, 95% CI = 0.27–0.77). Simpson’s index of diversity was 0.845 (95% CI 0.836–0.854) for the HDN trap and 0.778 (95% CI 0.769–0.787) for HLC, indicating that the HDN collected a greater diversity of mosquito species than HLC. Both HLC and HDN can distinguish between low and high biting rates and are crude ways to measure human-biting rate. The HDN is a simple and cheap method to estimate the human-biting rate outdoors without exposing collectors to mosquito bites.

## Introduction

Estimating the human-biting rate, the number of mosquito bites per person per day or night, is a key metric used for quantifying the risk of infection with mosquito-borne pathogens. Developing methods for estimating human-biting rates that do not expose collectors to vector-borne pathogens has been a major challenge in vector ecology, especially for species biting outdoors[1, 2]. Despite the development of innovative trapping methods the traditional human landing catch (HLC) method, where mosquitoes are collected when landing on exposed limbs, is still considered the ‘gold standard’[1, 3–5]. The strength of the HLC method is also its weakness as participants are exposed to potentially infective mosquito bites while performing catches. HLC can expose participants to diseases such as dengue for which no chemoprophylaxis or sterilising vaccine exists. Furthermore, whilst collectors can be protected from malaria using chemoprophylaxis[6], this method cannot be used where *Plasmodium* strains are less sensitive to antimalarials[7]. Alternative mosquito collection methods are necessary.

Here we set out to find an alternative sampling technique for collecting outdoor mosquitoes in Lao PDR. In the first experiment we compared the human-baited double net trap (HDN), CDC light trap, BG Sentinel trap and Suna trap. The HDN[1, 8] consists of two box nets; one protecting the collector and a second larger net which is placed directly over the inner net. The outer net is raised off the ground so that mosquitoes attracted to the human-bait are collected between the two nets. CDC light traps have been used for outdoor mosquito surveillance in Asia[9–13], though their primary use has been for estimating indoor-biting rates[3, 14, 15]. The BG sentinel trap releases artificial host-odours and employs attractive visual cues to attract outdoor-biting *Aedes* mosquitoes and is routinely used for surveillance [16–21]. The newly developed odour-baited Suna trap is effective at sampling *Anopheles gambiae* mosquitoes outdoors in Kenya[22]. In the second experiment the trap collecting the highest number of mosquitoes was compared directly against HLC to determine whether an alternative to the current ‘gold standard’ could be found.

## Materials and Methods

### Study area

The study was conducted during the middle and end of the rainy season in Luang Prabang province, Lao PDR. The area has a tropical monsoon climate with one hot, rainy season from May to October. During our study period temperatures ranged between 14.6°C-34.5°C with a relative humidity of 21.8%-100%. The 2013 annual rainfall in our study area was 1746.26 mm. For experiment 1 the teak plantation (19°41’09.19”N 102°07’13.84”E) and the primary school (19°41’08.27”N 102°07’12.99”E) both bordering Thin-Keo village were chosen for day and night collections respectively. For experiment 2 the secondary forest next to Silalek village (19°37’04.57”N 102°03’27.67”E) was chosen for day collections and the primary school in Thin-Keo village for night collections.

### Ethical statement

The study was approved by the ethical committee of the Ministry of Health in Lao PDR (approval number 017/NECHR issued 04-21-2013) and the School of Biological and Biomedical Sciences Ethics Committee, Durham University (issued 25-07-2013). Human landing catches were also approved by the Comité de Recherche Clinique de l'Institut Pasteur (approval number 2014–19 issued 08-07-2014).

### Study participants

Before starting the study verbal informed consent was provided by village leaders. Participants conducting HLCs and HDNs gave written, informed consent for their participation. Thirty-six healthy participants, males and females between 18–55 years old, were paid for their participation. Participants were given the opportunity to receive vaccination against Japanese encephalitis free of charge and were offered free medical treatment when they showed any symptoms suspected to be caused by mosquito-borne diseases. The study took place in an area without malaria. Human Landing Catches were conducted when dengue transmission was low in our study area, according to information provided by the Ministry of Health, Lao PDR.

### Mosquito sampling methods

#### Human-baited double net trap

**Fig 1:**
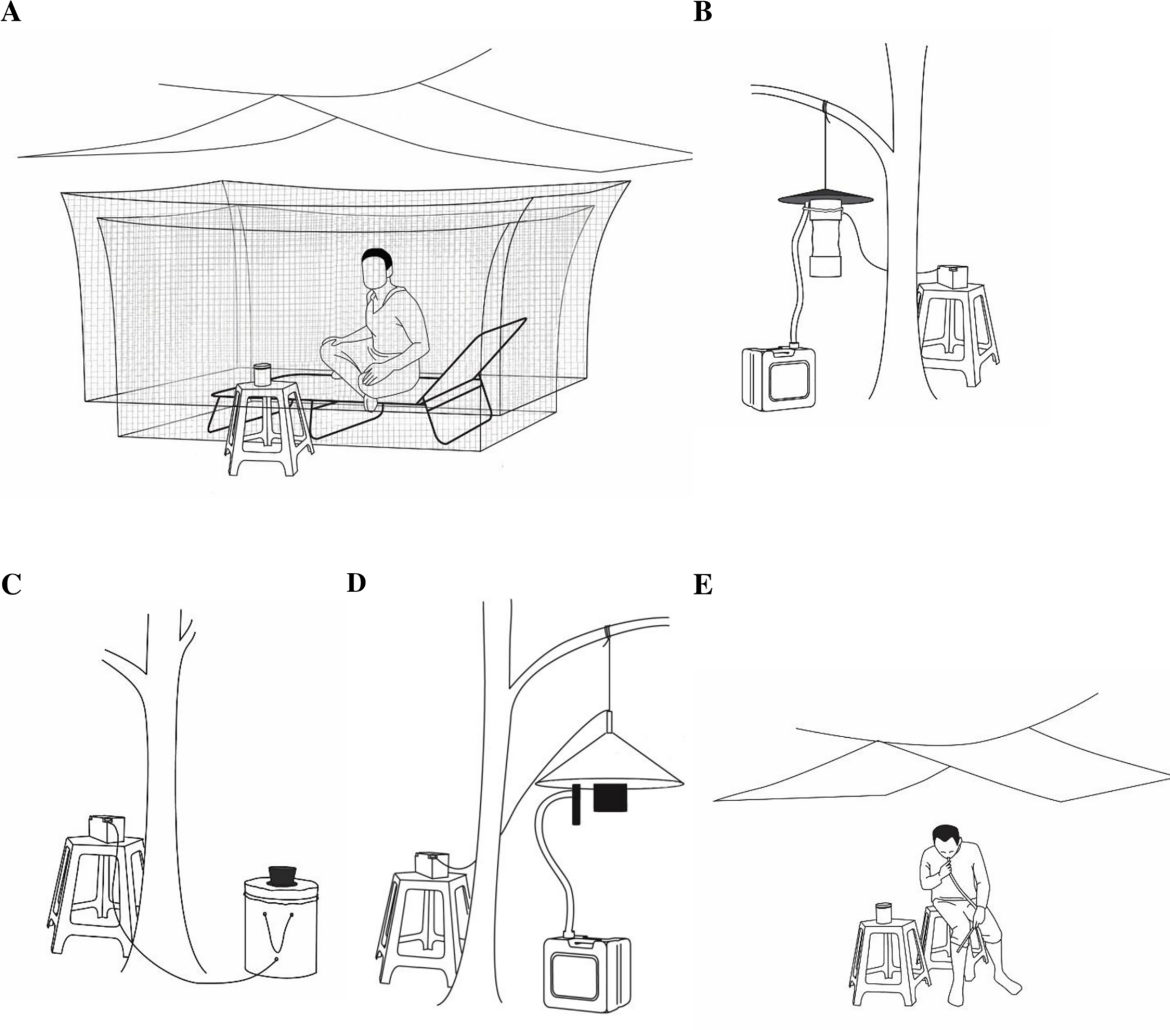
Mosquito sampling methods. (A) Human-baited double net trap with collecting cup (B) Odour-baited CDC light trap connected to a 6V battery with CO_2_-produced by sugar fermentation in the attached jerry can (C) Odour-baited BG sentinel trap connected to a 12V battery (D) Odour-baited Suna trap connected to a 12V battery with CO_2_-produced by sugar fermentation in the attached jerry can (E) Human Landing Catch method with collecting cup.

During catches performed with HDNs one adult occupied one trap and according to the different experiments collected mosquitoes for six or eight hours. Participants rested on a metal-framed bed with fabric inlay (20 cm high x 200 cm long x 70 cm wide) and were fully protected from mosquitoes by a small blue polyester bed net (97 cm high x 200 cm long x 100 cm wide, mesh size 1.5 mm) which was not treated with any insecticide and which hung over the bed to the ground. A larger untreated bed net (100 cm high x 250 cm long x 150 cm wide, mesh size 1.5 mm) which was also not treated with insecticide was hung over the smaller net and was raised 30 cm above the ground[1, 5]. Mosquitoes were caught in the ±20 cm gap between the two nets. Both nets were protected from the elements by plastic-sheeting roofs (Fig 1A). For 10 minutes of every hour participants raised the bottom of the inner net and aspirated mosquitoes caught between the nets into paper-cups. Mosquito catches of each hour were aspirated into different paper cups. When participants were not collecting mosquitoes, they rested inside the inner net. Participants had access to a stopwatch to monitor the time. Every collection day two field supervisors verified the participants conducted the collections as instructed.

#### CDC Light traps

CDC light traps (model 1912, John W. Hock Company, USA) used with the supplied incandescent bulb were suspended from trees with the lightbulb 1.5 m above the ground (Fig 1B). Mosquitoes attracted to the trap were sucked into the collection container by a 6V (6Ah) battery-powered fan. Carbon dioxide (CO_2_) produced by fermentation of sugar with yeast was supplied to the trap and one human-scented sock acted as an attractant[23]. CO_2_ was produced by mixing 250 g sugar (SPOON, Kasetphol sugar ltd, Thailand), 17.5 g yeast (Saf instant, Le saffre, Thailand) and 2.5 L water in a clean plastic jerry-can one hour before trapping[24]. The CO_2_ produced, passed along tubing (2 cm diameter) and was released at the trap entrance. Human-scented nylon socks, worn by one local volunteer for 24 hrs, were hung next to the trap entrance and replaced after eight days[25, 26]. When not in use the socks were stored in unused glass bottles at -20°C in order to preserve the human odour.

#### BG sentinel trap

The BG sentinel trap (Model 7.5, Biogents, Germany) is an odour-baited counter-flow trap comprised of a collapsible tubular container (40 cm high x 36 cm diameter) placed on the ground with the trap mouth opening upwards. The trap mouth opening is positioned 40 cm above the ground. Air is drawn into this black funnel trap opening (10 cm diameter), which then passes across the solid BG-lure® inside the main body and is forced out of the trap top through the gauze surrounding the funnel. Air movement is created by a fan powered by a 12V battery (11Ah) (Fig 1C). Mosquitoes attracted to the trap passed through the funnel and were collected in a gauze bag attached internally to the black funnel. As we could not identify previous studies that used CO_2_ produced by sugar fermentation as an attractant for BG sentinel traps, this was not included in our set-up.

#### Suna trap

The Suna trap (Biogents, Germany) is an odour-baited counter-flow trap with the trap mouth opening downwards. The trap was suspended 1.5 m above the ground, with the funnel opening set 1.0 m above the ground. The trap was powered by a 12V battery (11 Ah). At the base of the conical trap (52 cm high x 39 cm diameter) air was drawn into the trap through a black funnel (10 cm diameter). A synthetic blend of attractants mimicking human skin odours, impregnated on to nylon strips hanging in the cone of the trap, were blown out of the trap base through a perforated plastic cover surrounding the funnel entrance[27]. A jerry-can producing CO_2_, prepared as described above, was connected to the trap via a CO_2_ release tube located near the trap entrance (Fig 1D). Mosquitoes attracted to the trap passed through the trap funnel and were collected in a collection bag attached to the funnel inside the trap.

#### Human Landing Catches

HLC were conducted by 32 adults who collected mosquitoes landing on their exposed legs with an aspirator. Participants were involved in the comparison study for four days, of which two days were spend conducting the HLC method. One participant collected mosquitoes for eight hours. Collections were performed for 50 minutes every hour with a 10 minute break[3]. During collection participants sat on a 40 cm high stool and were protected from the elements by plastic-sheeting roofs (Fig 1E). Mosquitoes were aspirated from the exposed legs and collected in paper cups covered with netting.

### Sample size considerations

The first experiment was designed to detect whether HDN collected >50% more mosquitoes than other type of trap. Preliminary HDN data from study area (n = 12 days, mean no. mosquitoes/12 hrs = 48, standard deviation = 23) showed that 15 replicates (ω = 0.8, α = 0.05) of 12 hrs were necessary[28]. This sample size was increased to 20 catching occasions. For the second experiment a minimum correlation of 0.44 between the best trap and HLC was considered acceptable. Sample size calculations suggested 30 replicates were needed to obtain this (ω = 0.8, α = 0.05)[28], which was increased to 32 for a balanced design.

### Human-baited double net trap and outdoor traps comparison (experiment 1)

In July 2013 comparisons were made between the HDN, CDC light trap, BG sentinel trap and Suna trap in teak plantations for day collections and at a primary school for night collections. All collections were conducted outdoors. At each site there were two parallel transects, 30 m apart. On each transect four traps were placed, one of each trap type, positioned 5 m apart (n = 4 traps/transect). Four participants conducted the HDN method, rotated randomly between the four transects (two transects/day and night) and changed every six hours. If a single trap malfunctioned all collections from that transect were discarded and the experiment repeated. Day collections were made for 10 days from 07.00 to 19:00 h and night collections for 10 nights from 19:00 to 07:00 h (i.e. 20 day and 20 night comparisons)

### Human landing catch and human-baited double net trap comparison (experiment 2)

Collections were made during September and October 2014 in the secondary forest from 10:00 to 18:00 h and at the primary school from 17:00 to 01:00 h. All collections were conducted outdoors. HLC and HDN collections were made 5 m apart, and then duplicated 50 m away (i.e. four traps per occasion). This set up was repeated 16 times for day collections and 16 times for night collections (total of 64 comparisons between HLC-HDN). A group of four participants were randomly assigned to one of the four locations for four days or nights, so that variation in attractiveness between collectors was compounded with location attractiveness. After four days/nights of collection the participant group was changed for a group of four new participants. While participants were linked to a location, traps were rotated between locations using a 4 x 4 Latin-square design. The Latin-square design was repeated eight times (n = 32 comparison days) to ensure that the location variation, trapping ability of each participant and odour of each participant would not be associated with one trapping method. Thus in total 32 participants took part in the comparison, collecting mosquitoes using both the HLC and HDN method.

### Mosquito identification

Mosquitoes were morphologically identified to species complex using stereo-microscopes and recognized keys of the Indochinese region[29].

### Statistical analysis

Analyses of sampling efficiency for both experiments were performed using general linear modelling with a negative binomial model for count data and a log-link function (IBM SPSS statistics, ver. 20). Species diversity was compared for day and night collections using Simpson’s index of diversity (1-D) with results representing diversity from 0 (no diversity) to 1 (infinite diversity)[30, 31].

## Results

### Human-baited double net trap and outdoor traps comparison (experiment 1)

Overall 1144 female mosquitoes (978 HDN, 35 CDC light trap, 102 BG sentinel trap, 29 Suna trap) belonging to 48 species (45 species HDN, 16 species CDC light trap, 20 species BG sentinel trap, 13 species Suna trap) were collected. Seven mosquitoes could not be identified. Of the total number of female mosquitoes collected 31.2% (357/1144) were *Aedes* mosquitoes, with *Ae*. *albopictus* most abundant (51.8%, 185/357), 23.3% (267/1144) were *Anopheles* mosquitoes, with *An*. *barbumbrosus* most abundant (73.4%, 196/267) and 16.3% (188/1144) were *Culex* mosquitoes, with *Cx*. *vishnui* most common (40.4%, 76/188). About 30% of sampling occasions yielded no mosquitoes (0% of HDN, 65% of CDC light trap, 2.5% of BG sentinel trap, 52.5% of Suna trap).

Mosquito numbers varied significantly between traps, but not by location or collection date (Table 1). Overall HDN traps caught 34.48 (95% Confidence Interval, CI 18.87–66.67) times more mosquitoes than CDC light traps, 10.99 (95% CI 6.54–18.52) times more than BG sentinel traps and 43.48 (95% CI 22.72–76.92) times more than Suna traps (detailed information Table 2). More *Aedes* mosquitoes, including *Aedes albopictus*, *Anopheles* mosquitoes and *Culex* mosquitoes were collected by the HDN traps than the three other traps during both the day and the night.

**Table 1:** Analysis of female mosquitoes collected by the human-baited double net trap and outdoor traps comparison (experiment 1).

		Catch size	Location	Date	Trap type
	Time of day	Mean (95% CI)	*P*	*P*	*P*
**Total mosquitoes**	**Day**	8.46 (5.47–11.45)	0.742	0.372	<0.001*
	**Night**	5.84 (3.54–8.13)	0.248	0.104	<0.001*
***Aedes* mosquitoes**	**Day**	3.91 (2.50–5.33)	0.902	0.540	<0.001*
	**Night**	0.55 (0.12–0.98)	0.508	0.070	0.002*
***Aedes albopictus***	**Day**	2.23 (1.37–3.08)	0.954	0.871	<0.001*
***Anopheles* mosquitoes**	**Night**	3.06 (1.71–4.41)	0.302	0.203	<0.001*
***Culex* mosquitoes**	**Night**	2.06 (1.12–3.00)	0.527	0.528	<0.001*

Results are shown for day (n = 16) and night (n = 16) collections, for all locations (n = 8), for all collection dates (n = 8) and for all trap types (n = 4). As the catch sizes were too low, no night values for *Aedes albopictus* and no day values for *Anopheles* and *Culex* mosquitoes are shown. CI, Confidence Interval.

*significantly different, *P*<0.05.

**Table 2:** Means and rate ratio of female mosquitoes collected by the human-baited double net trap and outdoor traps comparison (experiment 1).

		HDN		CDC light trap			BG sentinel trap			Suna trap		
	Time of day	Mean catch size (95% CI)	RR (95% CI)	Mean catch size (95% CI)	RR (95% CI)	*P*	Mean catch size (95% CI)	RR (95% CI)	*P*	Mean catch size (95% CI)	RR (95% CI)	*P*
**Total mosquitoes**	**Day**	28.65 (22.71–34.59)	**1**	0.15 (-0.02–0.32)	0.005 (0.001–0.02)	<0.001*	4.25 (3.17–5.33)	0.14 (0.07–0.29)	<0.001*	0.80 (0.31–1.29)	0.02 (0.009–0.05)	<0.001*
	**Night**	20.25 (14.66–25.84)	**1**	1.60 (0.47–2.73)	0.06 (0.02–0.13)	<0.001*	0.85 (0.34–1.36)	0.03 (0.01–0.08)	<0.001*	0.65 (0.19–1.11)	0.03 (0.01–0.07)	<0.001*
***Aedes* mosquitoes**	**Day**	13.15 (10.12–16.18)	1	0.10 (-0.04–0.24)	0.007 (0.001–0.03)	<0.001*	2.15 (1.29–3.01)	0.14 (0.07–0.30)	<0.001*	0.25 (-.01–0.51)	0.02 (0.005–0.05)	<0.001*
	**Night**	1.75 (0.05–3.45)	1	0.05 (-0.05–0.15)	0.03 (0.003–0.29)	0.003*	0.30 (0.03–0.57)	0.09 (0.002–0.47)	0.004*	0.10 (-0.04–0.24)	0.04 (0.004–0.31)	0.002*
***Aedes albopictus***	**Day**	7.70 (5.75–9.65)	1	0.05 (-0.05–0.15)	0.006 (0.001–0.05)	<0.001*	1.0 (0.60–1.40)	0.12 (0.05–0.27)	<0.001*	0.15 (-0.02–0.32)	0.02 (0.004–0.06)	<0.001*
***Anopheles mosquitoes***	**Night**	11.00 (7.39–14.61)	1	1.15 (0.16–2.14)	0.06 (0.02–0.16)	<0.001*	0.10 (-0.11–0.31)	0.005 (0.001–0.03)	<0.001*	N/A	N/A	N/A
***Culex* mosquitoes**	**Night**	7.25 (4.48–10.02)	1	0.25 (0.04–0.46)	0.03 (0.01–0.09)	<0.001*	0.25 (-0.01–0.51)	0.03 (0.01–0.10)	<0.001*	0.50 (0.06–0.94)	0.05 (0.02–0.13)	<0.001*

Mosquito data are shown for day (n = 16) and night (n = 16) collections. As the catch sizes were too low, no night values for *Aedes albopictus* mosquitoes and no day values for *Anopheles* and *Culex* mosquitoes are depicted. CI, Confidence Interval; N/A, Not Applicable; RR, Rate ratio.

*significantly different, *P*<0.05.

### Human landing catch and human-baited double net trap comparison (experiment 2)

**Fig 2:**
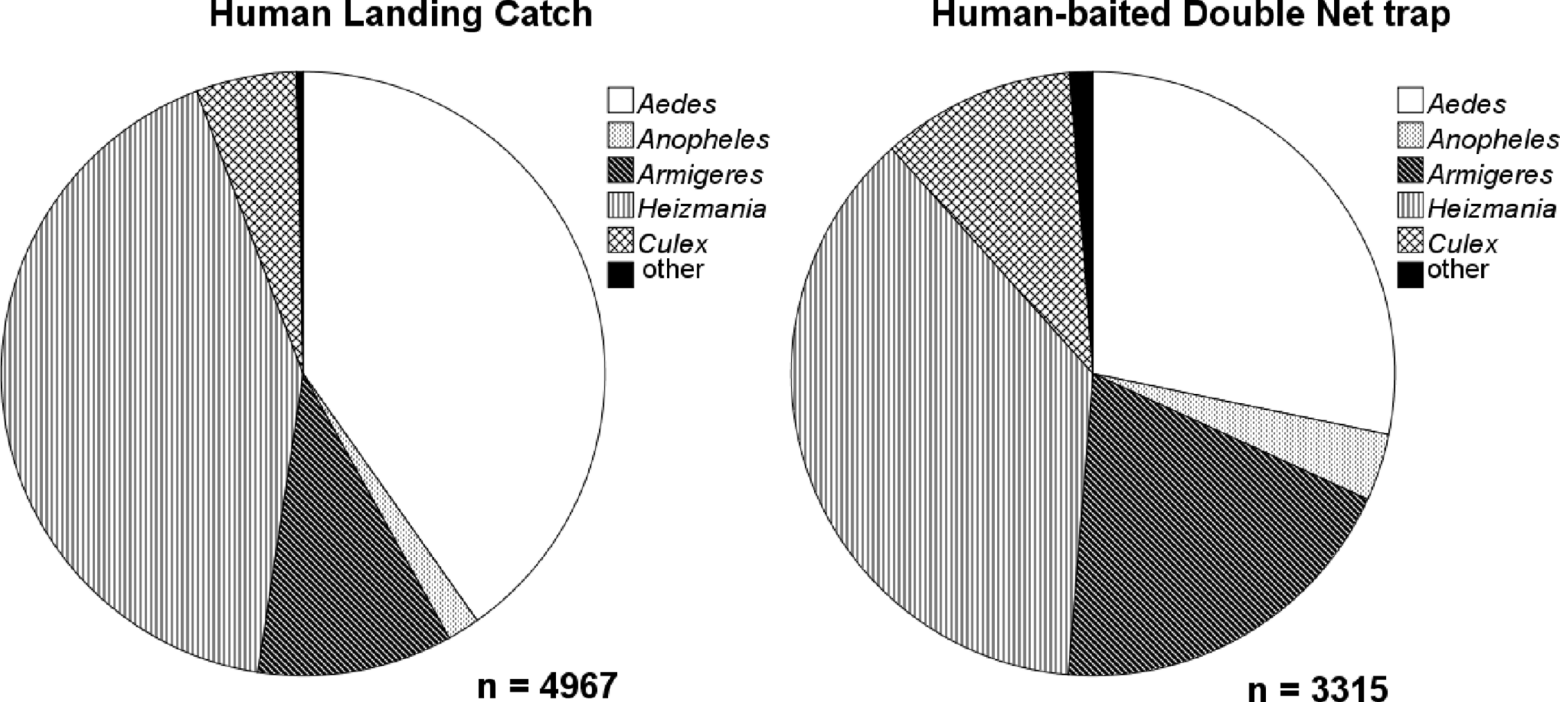
Species diversity of mosquitoes collected by the human landing catch method and human-baited double net traps (experiment 2).

**Fig 3:**
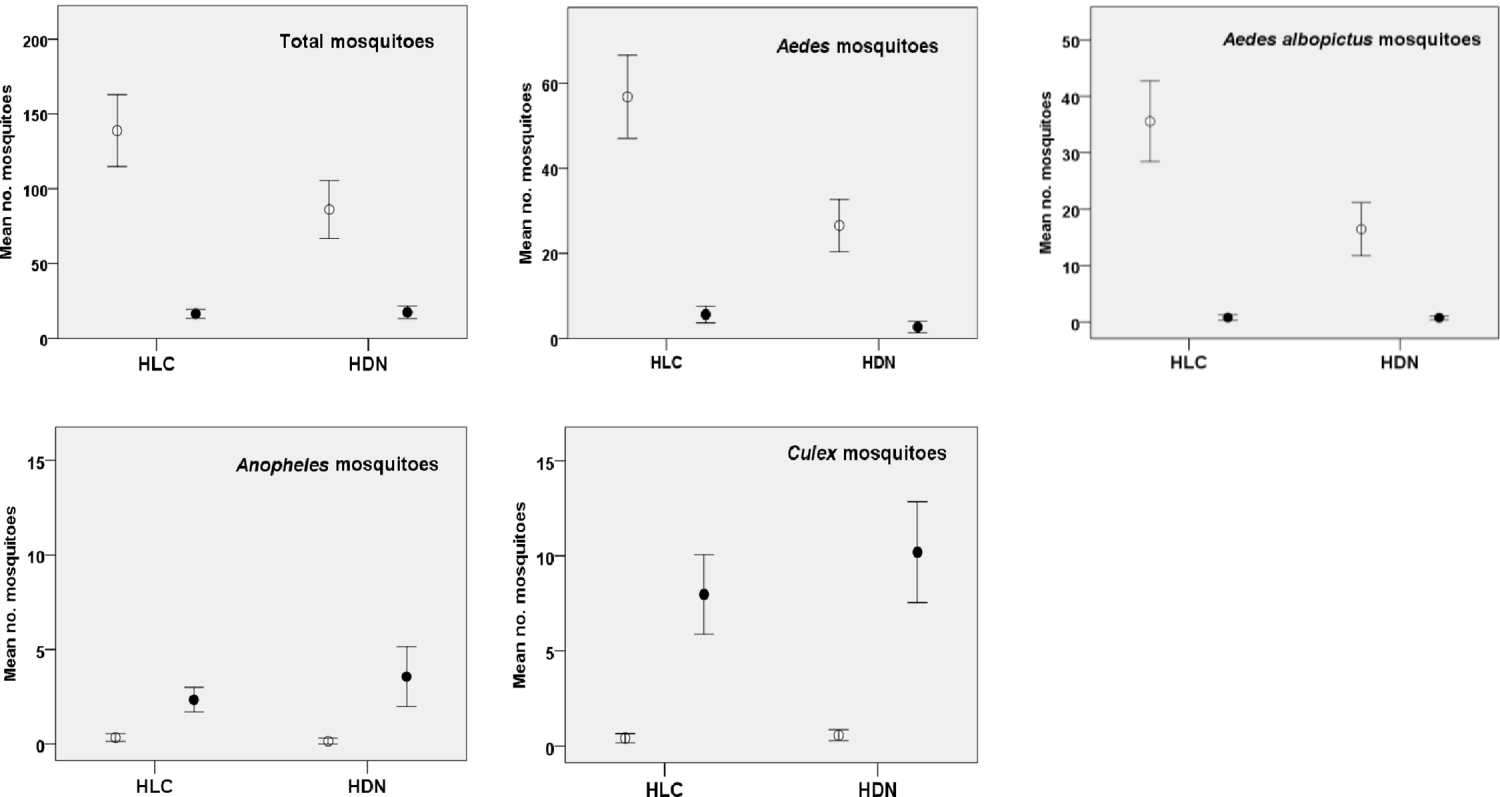
Means and confidence interval for female mosquitoes collected by the human landing catch and human-baited double net trap comparison (experiment 2) Female mosquitoes collected both day ○ and night ● with 95% confidence intervals. HDN, human-baited double net trap; HLC, human landing catch method.

A total of 8282 female mosquitoes (4967 HLC, 3315 HDN), belonging to 66 species (48 species HLC, 63 species HDN) were collected (Fig 2). From the total number of female mosquitoes collected, 39.9% were *Heizmania* species (3308/8282), with *Heizmania mattinglyi* most common (91.6%, 3029/3308), 35.4% were *Aedes* species (2934/8282), with *Ae*. *albopictus* most frequent (58.4%, 1714/2934), 2.5% *Anopheles* species (205/8282), with *An*. *barbumbrosus* most common (77.1%, 158/205) and 7.4% *Culex* species (612/8282), with *Cx*. *vishnui* most abundant (83.0%, 508/612) (Fig 3). Only 21 possible malaria vectors (*An*. *minimus*, *An*. *maculatus*, *An*. *dirus s*.*l*. and *An*. *barbirostris*) were collected. Nearly 20% of sampling occasions yielded no mosquitoes (21% HDN, 19% HLC).

**Fig 4:**
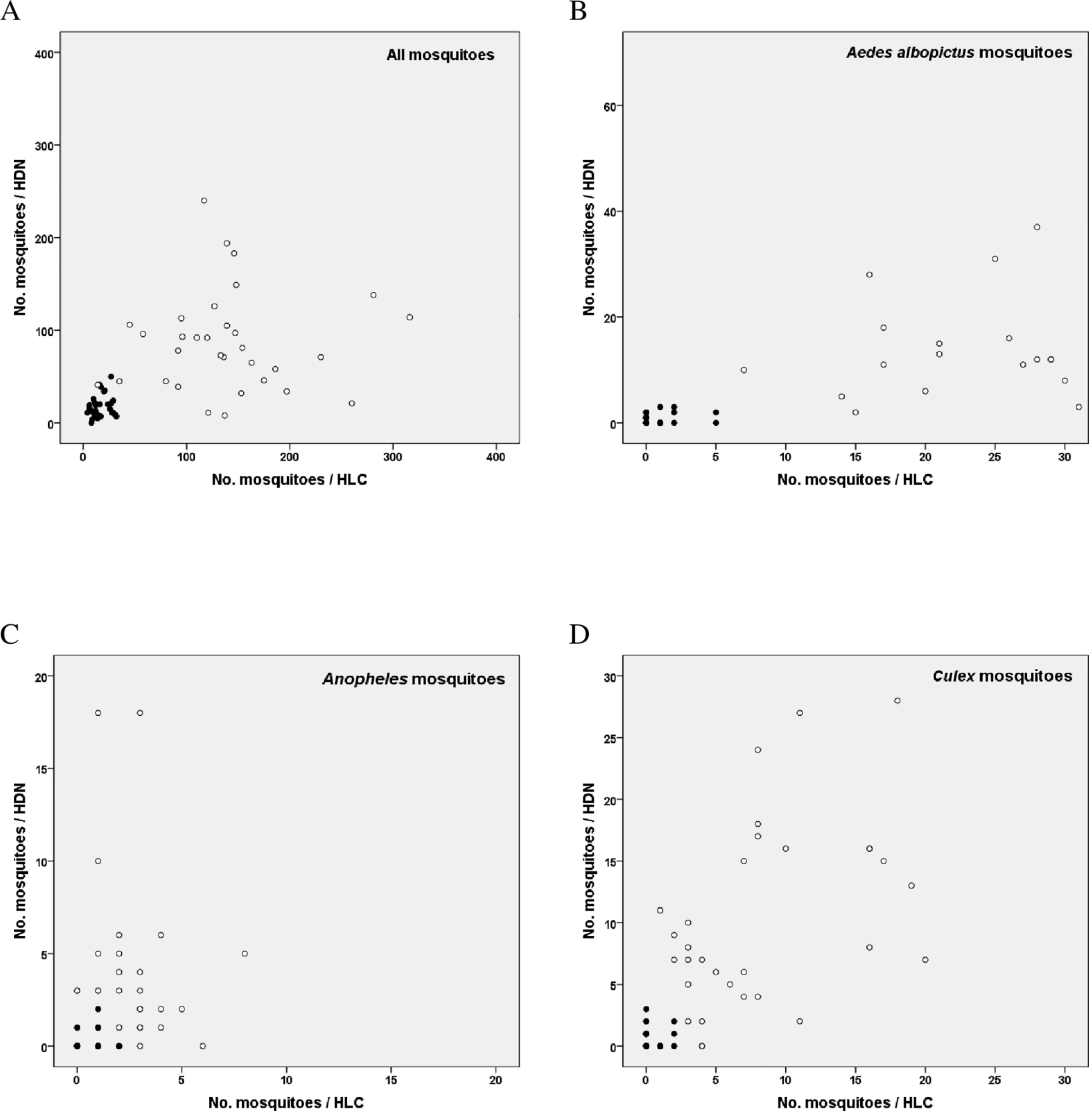
Comparison of the human landing catch and human-baited double net trap (experiment 2) Female mosquitoes collected both day ○ and night ● (A) All mosquito species collected by human landing collections (HLC) (n = 4967) and human baited double net (HDN) collections (n = 3315) (B) All *Aedes albopictus* mosquitoes collected by human landing collections (HLC) (n = 1163) and human baited double net (HDN) collections (n = 551) (C) All *Anopheles* mosquito species collected by human landing collections (HLC) (n = 86) and human baited double net (HDN) collections (n = 119) (D) All *Culex* mosquito species collected by human landing collections (HLC) (n = 268) and human baited double net (HDN) collections (n = 344).

The HDN trap collected similar number of mosquitoes of all species as HLC (Rate ratio, RR = 0.78, 95% CI 0.55–1.13, *P* = 0.186). However more detailed analysis showed that, whilst HDN collected a similar number of *Anopheles* and *Culex* mosquitoes when compared to HLC, the HDN method under-estimated the number of *Aedes* species, including the number of *Aedes albopictus*, by half (Table 3). Sampling was not affected by trap location nor date. The HDN and HLC collected similarly high number of mosquitoes during the day and similarly low number during the night (Fig 4). However no correlation was evident within the day collections or the night collections.

**Table 3:** Analysis of female mosquitoes collected by the human landing catch and human-baited double net trap comparison (experiment 2).

		Mean catch size		Location	Date	Trap	HDN
	Time of day	HLC(95% CI)	HDN(95% CI)	*P*	*P*	*P*	RR (95% CI)
**Total mosquitoes**	**Day**	138.81(114.74–162.89)	86.16(66.91–105.40)	0.637	0.941	0.054	0.60(0.36–1.01)
	**Night**	16.41(13.34–19.47)	17.44(13.24–21.64)	0.570	0.814	0.946	0.98(0.58–1.66)
***Aedes* species mosquitoes**	**Day**	56.78(47.01–66.55)	26.53(20.38–32.76)	0.741	0.968	0.003*	0.46(0.27–0.77)
***Aedes albopictus***	**Day**	35.56(28.42–42.71)	16.47(11.74–21.20)	0.817	0.938	0.003*	0.45(0.27–0.77)
***Anopheles* species mosquitoes**	**Night**	2.34(1.76–3.00)	3.56(1.98–5.15)	0.358	0.353	0.648	1.16(0.61–2.20)
***Culex* species mosquitoes**	**Night**	7.97(5.89–10.10)	10.18(7.53–12.84)	0.236	0.434	0.397	1.26(0.74–2.17)

Mosquito data are shown for total of day (n = 16) and night (n = 16) collections for all locations (n = 4), for all collection dates (n = 8) and for all trap types (n = 2). As the catch sizes were too low, no night values for *Aedes* and *Aedes albopictus* mosquitoes and no day values for *Anopheles* and *Culex* mosquitoes are depicted. CI, Confidence Interval; HDN, Human-baited Double Net; HLC, Human Landing Catches; RR, Rate Ratio.

*significantly different, *P*<0.05.

Species diversity calculated using Simpson’s index of diversity was 0.845 (95% CI 0.836–0.854) for the HDN collection method, which was higher than for the HLC method (1-D = 0.778 with 95% CI 0.769–0.787).

## Discussion

This study demonstrated that the HDN method is more efficient at collecting outdoor mosquitoes than CDC light traps, BG sentinel traps and Suna traps, and that it can be used as a more ‘ethical’ alternative to HLC. The low mosquito numbers collected by the traps not using human subject was disappointing, but perhaps not surprising. The suitability of CO_2-_baited CDC light traps for collecting outdoor mosquitoes is debatable and is expected to be highly dependent on mosquito species present[12, 13]. Furthermore this trap is likely more efficient at night due to the use of a light, with the trap collecting a higher number of mosquitoes at night than during the day. The BG sentinel trap is a tool used for routine mosquito surveillance of *Ae*. *albopictus* and *Ae*. *aegypti* in North America, Singapore and Australia[20, 21, 32]. The BG sentinel trap collected a higher number of mosquitoes during the day than the night, with the visual cues of the trap most likely involved in attracting the mosquitoes. Whilst in our study the BG sentinel trap did catch more *Aedes* species than both the CDC light trap and the Suna trap, numbers caught were still seven times less than HDN for day collection. The Suna trap has only been tested in one study in Kenya and the blend of attractants was developed based on host-seeking behaviour of *An*. *coluzzii* and *An*. *gambiae s*.*s*. mosquitoes, two African mosquitoes[22]. It is probable that skin odours from adults in Asia differ from those in Africa and consequently the Suna-blend is less attractive to Asian mosquitoes. In conclusion the CDC light trap, BG sentinel trap and Suna trap cannot be recommended for estimating outdoor human-biting rates in our study area.

Gater in 1935 appears to be the first to describe the use of the HDN method[33]. Since then HDN traps have been used in Africa, Asia and South America with varying success[1, 34–36]. This study showed that mosquito catches made with HDN and HLC are similar. Closer scrutiny of the data revealed this effect was due to sampling in two study areas, one with higher mosquito numbers than the other. Such an effect has been seen previously in comparisons between HLC and CDC light traps where an overall positive linear association was found between the two collection methods[37, 38]. In both cases when only low density data were considered no correlation was evident. Both HLC and HDN can distinguish between low and high mosquito densities.

In our study the HDN collected similar numbers of *Anopheles* and *Culex* mosquitoes as HLC, but under-estimated the number of *Aedes* mosquitoes. In earlier studies in Africa the HLC collected twice as many mosquitoes as human baited single bed net in Uganda[39], almost four times as many in Nigeria[40] and 7.5 times as many as a double net trap in Cameroon[34]. Concerns have been raised about HDN collections underestimating the true mosquito abundance as mosquitoes would escape the double net trap when they cannot feed[1, 34, 41]. In our study this concern was reduced by conducting hourly collections. However the HDN method still systematically underestimated the number of *Ae*. *albopictus* collected compared to the HLC method. This is presumably because mosquitoes either failed to enter the trap or did not persist for very long in the HDN. Nonetheless it should be recognised that both HDNs and HLCs are only proxy estimates of exposure. It is likely that HDNs slightly under-estimate biting rates, whilst HLCs over-estimate biting rates, since few people sit still all night exposing their limbs to mosquitoes. Human-biting rate estimates derived from both can be improved by increasing trap numbers to reduce variance. Our conclusion is that whilst both HLC and HDN can distinguish between low and high mosquito densities, they are both crude ways of estimating biting rates.

A slightly greater diversity of mosquito species were collected with the HDN trap compared with the HLC method. This suggests the HDN trap collects anthropophilic mosquitoes coming to feed, mosquitoes seeking shelter and mosquitoes entering the bed net accidentally. The HDN is therefore appropriate for identifying outdoor mosquito diversity in South-east Asia. It likely collects fewer, but nevertheless representative numbers, of anthropophilic mosquitoes compared to HLC.

One limitation of our study was that it was powered to explore whether there was a relationship between total mosquito numbers caught using HLC and HDN. In the future there is need to increase the sample size to explore the relationship between the catching efficiency of both methods when sampling *Aedes*, *Anopheles* and *Culex* species separately. It will be of interest to do additional studies to investigate if the HDN allows detection of seasonal variation and if the HDN method could be used as early warning for increases in disease transmission intensity. Moreover, we did not check whether any of the collected mosquitoes had fed on the participants under the nets, so we cannot rule out the possibility that some participants were bitten during these collections.

HDN can be used for sampling anthropophilic mosquitoes outdoors and is likely to work in similar settings in South-east Asia. It is a simple and cheap method for estimating human-biting rates. Most importantly this procedure is an ethically acceptable alternative to HLC as it protects individuals from exposure to mosquito bites directly. Further studies are nevertheless needed to confirm the catching efficiency of HDNs against single vector species in other parts of Asia and the tropics.

## Supporting Information

S1 Dataset Dataset for human-baited double net trap and outdoor traps comparison (experiment 1) and human landing catch and human-baited double net trap comparison (experiment 2). (XLSX)

## Abbreviations

BG: Biogents
CDC: Centers for Disease Control and Prevention
CI: Confidence Interval
HDN: Human-baited Double Net
HLC: Human Landing Catches
PDR: People’s Democratic Republic
RR: Rate Ratio
SD: Standard Deviation
WHO: World Health Organization
